# Supplementary material for: Comparative efficacy of various oral hygiene care methods in preventing ventilator-associated pneumonia in critically ill patients: A systematic review and network meta-analysis
Source: PLoS One. 2024 Dec 13;19(12):e0313057. doi: 10.1371/journal.pone.0313057 (PMC11642986; doi:10.1371/journal.pone.0313057)
Supplement: S1 Fig — (DOCX) [file pone.0313057.s002.docx]

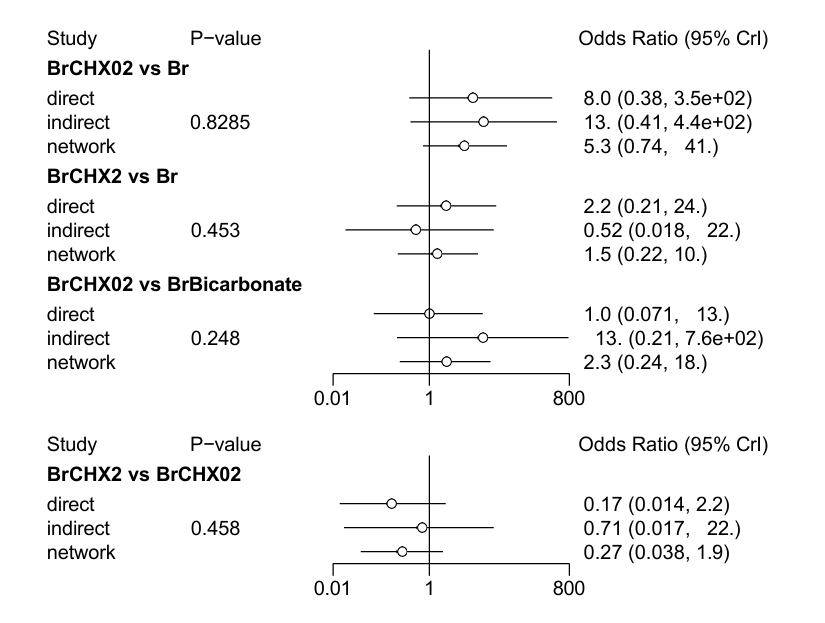


**S1 Fig. Forest plot for direct and indirect comparisons of treatment effects using the node-splitting method**

Br, brushing only; BrBicarbonate, brushing combined with bicarbonate; BrCHX02, brushing combined with chlorhexidine 0.2%; BrCHX2, brushing combined with chlorhexidine 2%.

The sources of data for each intervention: Br [32,33,35,42], BrBicarbonate [32,35], BrCHX02 [32,38,39], BrCHX2 [33,39]
