# Supplementary material for: Comparative efficacy of various oral hygiene care methods in preventing ventilator-associated pneumonia in critically ill patients: A systematic review and network meta-analysis
Source: PLoS One. 2024 Dec 13;19(12):e0313057. doi: 10.1371/journal.pone.0313057 (PMC11642986; doi:10.1371/journal.pone.0313057)
Supplement: S2 Fig — (DOCX) [file pone.0313057.s003.docx]

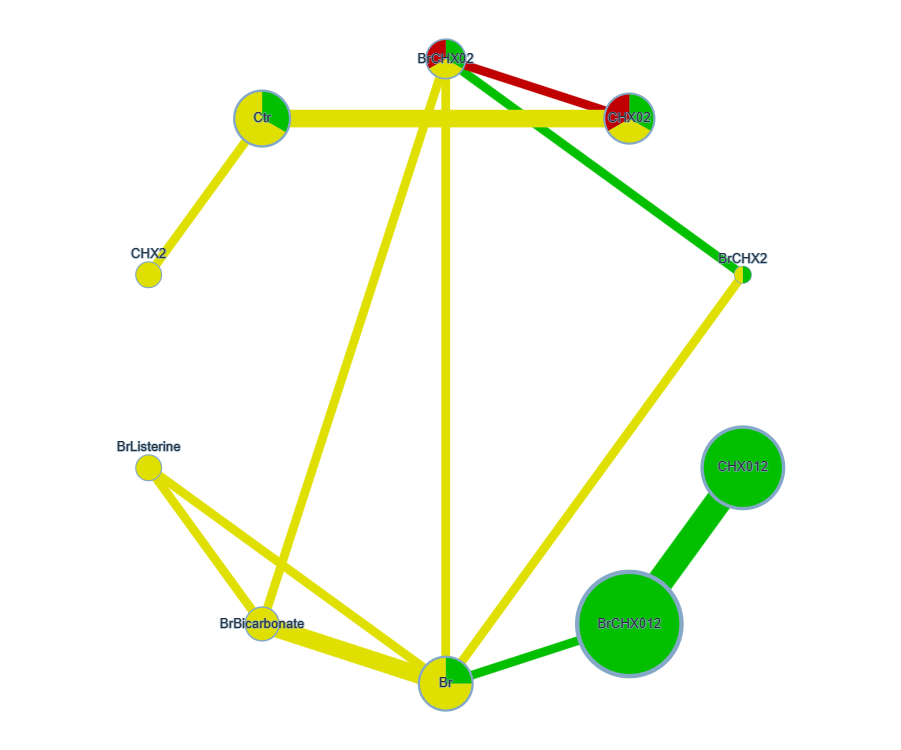


**S2 Fig. Network plots of oral hygiene care**

The width of the edges indicates the number of studies randomized in each comparison, and the size of the nodes indicates the total number of participants who received the intervention. The colors of edges and nodes refer to the risk of bias: low (green), moderate (yellow), and high (red).

Br, brushing only; BrBicarbonate, brushing combined with bicarbonate; BrCHX012, brushing combined with chlorhexidine 0.12%; BrCHX02, brushing combined with chlorhexidine 0.2%; BrCHX2, brushing combined with chlorhexidine 2%; BrListerine, brushing combined with Listerine; CHX012, chlorhexidine 0.12% only; CHX02, chlorhexidine 0.2% only; CHX2, chlorhexidine 2% only; Ctr, control group.

The sources of data for each intervention: Br [32,33,35,42], BrBicarbonate [32,35], BrCHX012 [31,34,40,42], BrCHX02 [32,38,39], BrCHX2 [33,39], BrListerine [35], CHX012 [31,34,40], CHX02 [30,36,38], CHX2 [37], Ctr [30,36,37].
