# Supplementary material for: Comparative efficacy of various oral hygiene care methods in preventing ventilator-associated pneumonia in critically ill patients: A systematic review and network meta-analysis
Source: PLoS One. 2024 Dec 13;19(12):e0313057. doi: 10.1371/journal.pone.0313057 (PMC11642986; doi:10.1371/journal.pone.0313057)
Supplement: S3 Fig — (DOCX) [file pone.0313057.s004.docx]

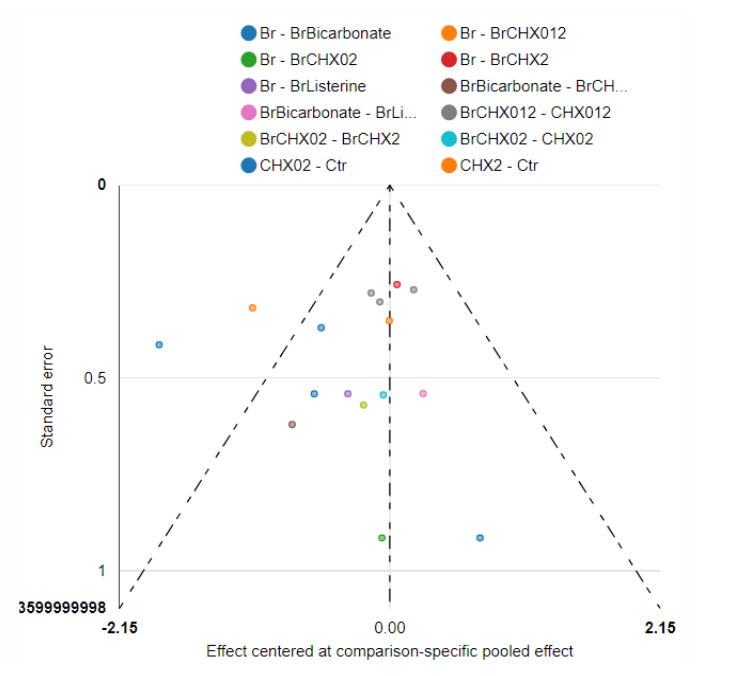


**S3 Fig. Funnel plot.**

Br, brushing only; BrBicarbonate, brushing combined with bicarbonate; BrCHX012, brushing combined with chlorhexidine 0.12%; BrCHX02, brushing combined with chlorhexidine 0.2%; BrCHX2, brushing combined with chlorhexidine 2%; BrListerine, brushing combined with Listerine; CHX012, chlorhexidine 0.12% only; CHX02, chlorhexidine 0.2% only; CHX2, chlorhexidine 2% only; Ctr, control group.

The sources of data for each intervention: Br [32,33,35,42], BrBicarbonate [32,35], BrCHX012 [31,34,40,42], BrCHX02 [32,38,39], BrCHX2 [33,39], BrListerine [35], CHX012 [31,34,40], CHX02 [30,36,38], CHX2 [37], Ctr [30,36,37].
