# Supplementary material for: Comparative efficacy of various oral hygiene care methods in preventing ventilator-associated pneumonia in critically ill patients: A systematic review and network meta-analysis
Source: PLoS One. 2024 Dec 13;19(12):e0313057. doi: 10.1371/journal.pone.0313057 (PMC11642986; doi:10.1371/journal.pone.0313057)
Supplement: S1 Text — (DOCX) [file pone.0313057.s005.docx]

**S1 Text. PubMed search strategy**

#1 "critical illness"[MeSH Terms]

#2 "critical illness"[Title/Abstract]

#3 "intensive care units"[MeSH Terms]

#4 "intensive care unit*"[Title/Abstract]

#5 "critical care"[MeSH Terms]

#6 "critical care"[Title/Abstract]

#7 "critically ill"[Title/Abstract]

#8 "ICU"[Title/Abstract]

#9 "respiration, artificial"[MeSH Terms]

#10 "mechanical ventilat*"[Title/Abstract]

#11 #1 OR #2 OR #3 OR #4 OR #5 OR #6 OR #7 OR #8 OR #9 OR #10

#12 "oral hygiene"[MeSH Terms]

#13 "oral"[Title/Abstract]

#14 "toothbrush*"[Title/Abstract]

#15 "mouth"[MeSH Terms]

#16 "mouth"[Title/Abstract]

#17 "dental care"[MeSH Terms]

#18 "dental"[Title/Abstract]

#19 "mouthwashes"[MeSH Terms]

#20 "mouthwash*"[Title/Abstract]

#21 #12 OR #13 OR #14 OR #15 OR #16 OR #17 OR #18 OR #19 OR #20

#22 "randomized controlled trial"[Publication Type]

#23 "controlled clinical trial"[Publication Type]

#24 "randomized"[Title/Abstract]

#25 "placebo"[Title/Abstract]

#26 "randomly"[Title/Abstract]

#27 "trial"[Title/Abstract]

#28 #22 OR #23 OR #24 OR #25 OR #26 OR #27

#29 "animals"[MeSH Terms] NOT "humans"[MeSH Terms]

#30 #28 NOT #29

#31 #11 AND #21 AND #30
