# Supplementary material for: Comparative efficacy of various oral hygiene care methods in preventing ventilator-associated pneumonia in critically ill patients: A systematic review and network meta-analysis
Source: PLoS One. 2024 Dec 13;19(12):e0313057. doi: 10.1371/journal.pone.0313057 (PMC11642986; doi:10.1371/journal.pone.0313057)
Supplement: S3 Table — (DOCX) [file pone.0313057.s008.docx]

**S3 Table. Assessment of quality of evidence using Confidence in Network meta-analysis (CINeMA)**

| Comparison | Number of studies | Within-study bias | Reporting bias | Indirectness | Imprecision | Heterogeneity | Incoherence | **Confidence rating** | Reason(s) for downgrading |
| --- | --- | --- | --- | --- | --- | --- | --- | --- | --- |
| Br:BrBicarbonate | 2 | Some concerns | Low risk | No concerns | Major concerns | No concerns | No concerns | **Very low** | ["Within-study bias","Imprecision"] |
| Br:BrCHX012 | 1 | No concerns | Low risk | No concerns | Major concerns | No concerns | No concerns | **Low** | ["Imprecision"] |
| Br:BrCHX02 | 1 | Some concerns | Some concerns | No concerns | No concerns | Major concerns | No concerns | **Very low** | ["Within-study bias","Reporting bias","Heterogeneity"] |
| Br:BrCHX2 | 1 | Some concerns | Low risk | No concerns | Major concerns | No concerns | No concerns | **Very low** | ["Within-study bias","Imprecision"] |
| Br:BrListerine | 1 | Some concerns | Low risk | No concerns | Major concerns | No concerns | No concerns | **Very low** | ["Within-study bias","Imprecision"] |
| BrBicarbonate:BrCHX02 | 1 | Some concerns | Low risk | No concerns | Major concerns | No concerns | No concerns | **Very low** | ["Within-study bias","Imprecision"] |
| BrBicarbonate:BrListerine | 1 | Some concerns | Low risk | No concerns | Major concerns | No concerns | No concerns | **Very low** | ["Within-study bias","Imprecision"] |
| BrCHX012:CHX012 | 3 | No concerns | Low risk | No concerns | Major concerns | No concerns | No concerns | **Low** | ["Imprecision"] |
| BrCHX02:BrCHX2 | 1 | No concerns | Low risk | No concerns | Major concerns | No concerns | No concerns | **Low** | ["Imprecision"] |
| BrCHX02:CHX02 | 1 | Major concerns | Low risk | No concerns | Major concerns | No concerns | No concerns | **Very low** | ["Within-study bias","Imprecision"] |
| CHX02:Ctr | 2 | No concerns | Low risk | No concerns | Major concerns | No concerns | No concerns | **Low** | ["Imprecision"] |
| CHX2:Ctr | 1 | Some concerns | Some concerns | No concerns | Major concerns | No concerns | No concerns | **Very low** | ["Within-study bias","Reporting bias","Imprecision"] |
| Br:CHX012 | 0 | No concerns | Low risk | No concerns | Major concerns | No concerns | No concerns | **Low** | ["Imprecision"] |
| Br:CHX02 | 0 | Some concerns | Low risk | No concerns | Major concerns | No concerns | No concerns | **Low** | ["Within-study bias","Imprecision"] |
| Br:CHX2 | 0 | Some concerns | Low risk | No concerns | Major concerns | No concerns | No concerns | **Very low** | ["Within-study bias","Imprecision"] |
| Br:Ctr | 0 | Some concerns | Low risk | No concerns | No concerns | Major concerns | No concerns | **Very low** | ["Within-study bias","Heterogeneity"] |
| BrBicarbonate:BrCHX012 | 0 | Some concerns | Low risk | No concerns | Major concerns | No concerns | No concerns | **Very low** | ["Within-study bias","Imprecision"] |
| BrBicarbonate:BrCHX2 | 0 | Some concerns | Low risk | No concerns | Major concerns | No concerns | No concerns | **Very low** | ["Within-study bias","Imprecision"] |
| BrBicarbonate:CHX012 | 0 | No concerns | Low risk | No concerns | Major concerns | No concerns | No concerns | **Low** | ["Imprecision"] |
| BrBicarbonate:CHX02 | 0 | Some concerns | Low risk | No concerns | Major concerns | No concerns | No concerns | **Very low** | ["Within-study bias","Imprecision"] |
| BrBicarbonate:CHX2 | 0 | Some concerns | Some concerns | No concerns | Major concerns | No concerns | No concerns | **Very low** | ["Within-study bias","Reporting bias","Imprecision"] |
| BrBicarbonate:Ctr | 0 | Some concerns | Low risk | No concerns | Major concerns | No concerns | No concerns | **Very low** | ["Within-study bias","Imprecision"] |
| BrCHX012:BrCHX02 | 0 | No concerns | Low risk | No concerns | No concerns | Major concerns | No concerns | **Low** | ["Heterogeneity"] |
| BrCHX012:BrCHX2 | 0 | No concerns | Low risk | No concerns | Major concerns | No concerns | No concerns | **Low** | ["Imprecision"] |
| BrCHX012:BrListerine | 0 | Some concerns | Low risk | No concerns | Major concerns | No concerns | No concerns | **Very low** | ["Within-study bias","Imprecision"] |
| BrCHX012:CHX02 | 0 | Some concerns | Low risk | No concerns | No concerns | Major concerns | No concerns | **Very low** | ["Within-study bias","Heterogeneity"] |
| BrCHX012:CHX2 | 0 | Some concerns | Low risk | No concerns | Major concerns | No concerns | No concerns | **Very low** | ["Within-study bias","Imprecision"] |
| BrCHX012:Ctr | 0 | No concerns | Low risk | No concerns | No concerns | Major concerns | No concerns | **Low** | ["Heterogeneity"] |
| BrCHX02:BrListerine | 0 | Some concerns | Low risk | No concerns | Major concerns | No concerns | No concerns | **Very low** | ["Within-study bias","Imprecision"] |
| BrCHX02:CHX012 | 0 | No concerns | Low risk | No concerns | Major concerns | No concerns | No concerns | **Low** | ["Imprecision"] |
| BrCHX02:CHX2 | 0 | Some concerns | Some concerns | No concerns | Major concerns | No concerns | No concerns | **Very low** | ["Within-study bias","Imprecision"] |
| BrCHX02:Ctr | 0 | Major concerns | Low risk | No concerns | Major concerns | No concerns | No concerns | **Very low** | ["Within-study bias","Imprecision"] |
| BrCHX2:BrListerine | 0 | Some concerns | Low risk | No concerns | Major concerns | No concerns | No concerns | **Very low** | ["Within-study bias","Imprecision"] |
| BrCHX2:CHX012 | 0 | No concerns | Low risk | No concerns | Major concerns | No concerns | No concerns | **Low** | ["Imprecision"] |
| BrCHX2:CHX02 | 0 | Major concerns | Low risk | No concerns | Major concerns | No concerns | No concerns | **Very low** | ["Within-study bias","Imprecision"] |
| BrCHX2:CHX2 | 0 | Some concerns | Some concerns | No concerns | Major concerns | No concerns | No concerns | **Very low** | ["Within-study bias","Imprecision"] |
| BrCHX2:Ctr | 0 | Some concerns | Low risk | No concerns | No concerns | Major concerns | No concerns | **Very low** | ["Within-study bias","Heterogeneity"] |
| BrListerine:CHX012 | 0 | No concerns | Low risk | No concerns | Major concerns | No concerns | No concerns | **Low** | ["Imprecision"] |
| BrListerine:CHX02 | 0 | Some concerns | Low risk | No concerns | Major concerns | No concerns | No concerns | **Very low** | ["Within-study bias","Imprecision"] |
| BrListerine:CHX2 | 0 | Some concerns | Low risk | No concerns | Major concerns | No concerns | No concerns | **Very low** | ["Within-study bias","Imprecision"] |
| BrListerine:Ctr | 0 | Some concerns | Low risk | No concerns | Major concerns | No concerns | No concerns | **Very low** | ["Within-study bias","Imprecision"] |
| CHX012:CHX02 | 0 | No concerns | Low risk | No concerns | Major concerns | No concerns | No concerns | **Low** | ["Imprecision"] |
| CHX012:CHX2 | 0 | No concerns | Low risk | No concerns | Major concerns | No concerns | No concerns | **Low** | ["Imprecision"] |
| CHX012:Ctr | 0 | No concerns | Low risk | No concerns | No concerns | Major concerns | No concerns | **Low** | ["Heterogeneity"] |
| CHX02:CHX2 | 0 | Some concerns | Some concerns | No concerns | Major concerns | No concerns | No concerns | **Very low** | ["Within-study bias","Imprecision"] |

Br, brushing only; BrBicarbonate, brushing combined with bicarbonate; BrCHX012, brushing combined with chlorhexidine 0.12%; BrCHX02, brushing combined with chlorhexidine 0.2%; BrCHX2, brushing combined with chlorhexidine 2%; BrListerine, brushing combined with Listerine; CHX012, chlorhexidine 0.12% only; CHX02, chlorhexidine 0.2% only; CHX2, chlorhexidine 2% only; Ctr, control group.

The sources of data for each intervention: Br [32,33,35,42], BrBicarbonate [32,35], BrCHX012 [31,34,40,42], BrCHX02 [32,38,39], BrCHX2 [33,39], BrListerine [35], CHX012 [31,34,40], CHX02 [30,36,38], CHX2 [37], Ctr [30,36,37].
